# Simultaneous occurrence of two distinct histotypes of ovarian endometriosis-associated cancer in bilateral ovaries: implications for monoclonal histogenesis from a case report

**DOI:** 10.3389/fonc.2023.1280529

**Published:** 2023-11-27

**Authors:** Luyang Zhao, Yunyue Liu, Feng Liu, Xiangshu Jin, Jia Xu, Shuting Zhou, Yuanguang Meng, Aijun Liu, Weng Yang

**Affiliations:** ^1^ Department of Obstetrics and Gynecology, The Seventh Medical Centre of Chinese People’s Liberation Army General Hospital, Beijing, China; ^2^ Department of Pathology, The Seventh Medical Centre of Chinese People’s Liberation Army (PLA) General Hospital, Beijing, China

**Keywords:** endometriosis-associated ovarian cancer, clear cell ovarian cancer, endometrioid ovarian cancer, endometriosis, eutopic endometrium

## Abstract

**Background:**

Transformation of endometriosis to malignancy is a rare occurrence. Clear cell ovarian cancer and endometrioid ovarian cancer are the two histotypes most consistently linked to endometriosis. The exact pathways leading to malignant transformation of endometriosis remain elusive.

**Case presentation:**

A 41-year-old woman presented to our hospital with a ten days history of abdominal pain which was not responsive to medication. Pathological examination revealed an unexpected finding of bilateral endometriosis associated with distinct malignancies: a clear cell carcinoma in the right ovary and a well-differentiated endometrioid carcinoma in the left ovary. Molecular analysis indicated a shared somatic driver mutation in ING1 in the eutopic endometrium and the bilateral ovaries while simultaneously exhibiting specific genetic alterations unique to each carcinoma. Notably, several common mutation sites were also identified, including previously reported common oncogenes (KRAS, PIK3CA, ARID1A). This finding prompts the hypothesis of a possible monoclonal origin of the two tumours.

**Conclusion:**

This case represents an exceedingly rare occurrence of two different histotypes of ovarian endometriosis-associated cancer manifesting simultaneously in bilateral ovaries. Based on genetic analysis, we hypothesize that these malignancies may have a monoclonal origin, providing insights into understanding the different biological mechanisms underlying carcinogenesis.

## 1 Introduction

Endometriosis is a chronic and progressive inflammatory disease that affects 10% of women in their reproductive years (1). Previous research has indicated a significant link between endometriosis and increased risk of clear cell ovarian cancer (CCOC) and endometrioid ovarian cancer (EOC), with risks elevated by 3.4-fold and 2.3-fold, respectively (2). Although transformation of endometriosis to malignancy is uncommon, occurring in only approximately 0.7-1.6% of women (3), recent robust epidemiological studies have raised questions about the accuracy of these rates. Approximately one-third of all CCOC and EOC cases are now believed to originate from endometriosis (4). Nonetheless, the precise carcinogenic pathways underlying transformation of endometriosis to malignancy remain unclear. An increasing number of clinicopathological studies have suggested the existence of distinct pathways for malignant evolution of endometriosis-associated CCOC and EOC (4). Here, we describe a rare case involving a 41-year-old female patient with simultaneous bilateral tumours, with the right ovary showing primary CCOC and the left ovary EOC. This case provides evidence of a monoclonal origin for the different histotypes.

## 2 Case description

A 41-year-old woman, gravida 2 and para 1, presented with a ten days history of abdominal pain which was not responsive to medication. The patient had regular menstrual periods without significant dysmenorrhoea. Bilateral ovarian cysts were detected during a routine physical examination approximately one year prior. However, ten days before admission to a local hospital, she had persistent lower abdominal pain without any apparent reason. Notably, some serum tumour markers showed remarkable elevation: carbohydrate antigen 125 (CA125) of 1229 U/ml (normal range, 0–35), carbohydrate antigen 199 (CA199) of 7107 U/ml (normal range, 0–35) and carcino-embryonic antigen (CEA) 44.1 U/ml (normal range, 0–11). Conversely, carbohydrate antigen 153 and human epididymis protein 4 showed no significant increase. Abdominal computed tomography revealed a 15*10 cm cystic-solid tumour. Given the elevated white blood cell count of 19.35*10^9/l (with a neutrophil count of 93.2%), the local hospital initiated a one-week course of anti-infective treatment, which partially alleviated her symptoms. Her serum tumour marker levels decreased slightly, as follows: CA125 710 U/ml, CA199 2998 U/ml, and CEA 25.98 U/ml. Besides, she had no other medical conditions and she denied a family history of endometriosis or cancer. Subsequently, the patient sought further treatment at our hospital.

**Figure 1:**
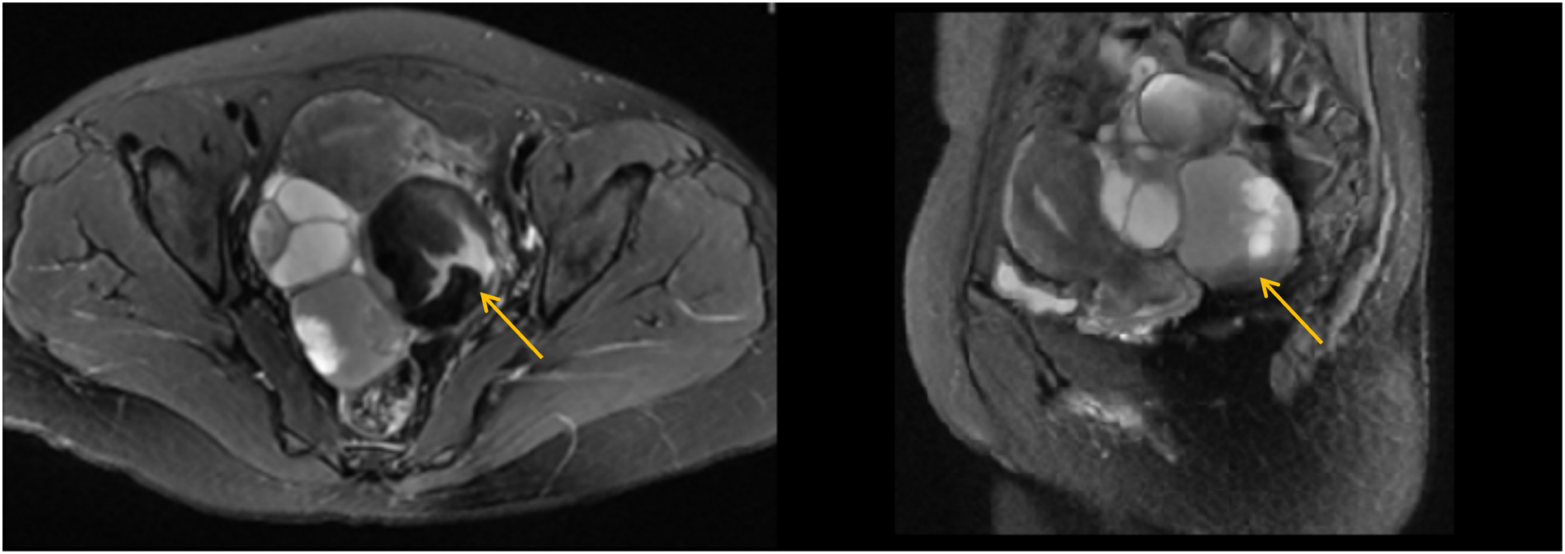
Magnetic resonance imaging (MRI) of the pelvic cavity (T2-weighted image). The MRI suggested potential malignancy due to multiple cystic-solid masses (the largest one 7.6*6.6cm, yellow arrow) originating from the adnexal region, accompanied by intra-cyst bleeding.

Physical examination showed that her body mass index was 21.3 kg/m2 (height: 165 cm, weight: 58 kg), with no significant recent changes. Abdominal assessment revealed slight pressure pain without obvious rebound pain in the lower abdomen. Gynaecological examination indicated a normally sized uterus with limited mobility. A tender, solid-cystic mass measuring 12 cm in diameter was noted posterior to the uterus. No palpable nodules were found on palpation of the anus. Her white blood cell count was within the normal range in our laboratory analysis. CA125 levels decreased to 417 U/ml, and CA199 levels decreased to 1772 U/ml. Gastroenteroscopy yielded normal results. Transvaginal ultrasonography revealed a 12*11*4.6 cm cystic-solid mass in the posterior uterus displaying a nonhomogeneous echo. The magnetic resonance imaging suggested potential malignancy due to multiple cystic-solid masses originating from the adnexal region accompanied by intracyst bleeding (**Figure 1**).

Robotic surgery was performed to explore the abdominal pelvic lesions. Chocolate-like discoloured deposits were distributed in the abdominal cavity, primarily within the greater omentum and peritoneal mesentery. The bilateral adnexa were adherent to the pelvic wall. The left ovary showed a mass of approximately 10*10 cm and the right ovary a mass of approximately 6*5 cm, both containing chocolate-like fluid and several papillary solid protrusions. Frozen pathology analysis of the left ovarian cyst suggested a well-differentiated carcinoma. Subsequently, a comprehensive surgical intervention, including total hysterectomy, bilateral salpingo-oophorectomy, omentectomy and lymphadenectomy, was performed. The final diagnosis was International Federation of Gynaecology and Obstetrics (FIGO) stage IC1, with a grade 2 endometrioid adenocarcinoma in the left ovary and a FIGO stage IC1 clear cell carcinoma in the right ovary (elaborated pathology description provided below). The patient underwent a course of six chemotherapy cycles with paclitaxel-albumin and carboplatin, which resulted in complete relief. Postsurgery, her serum CA125 level decreased to 96.3 U/ml and the CA199 level to 239.7 U/ml. All serum biomarkers returned to normal levels after the second cycle of chemotherapy.

**Figure 2:**
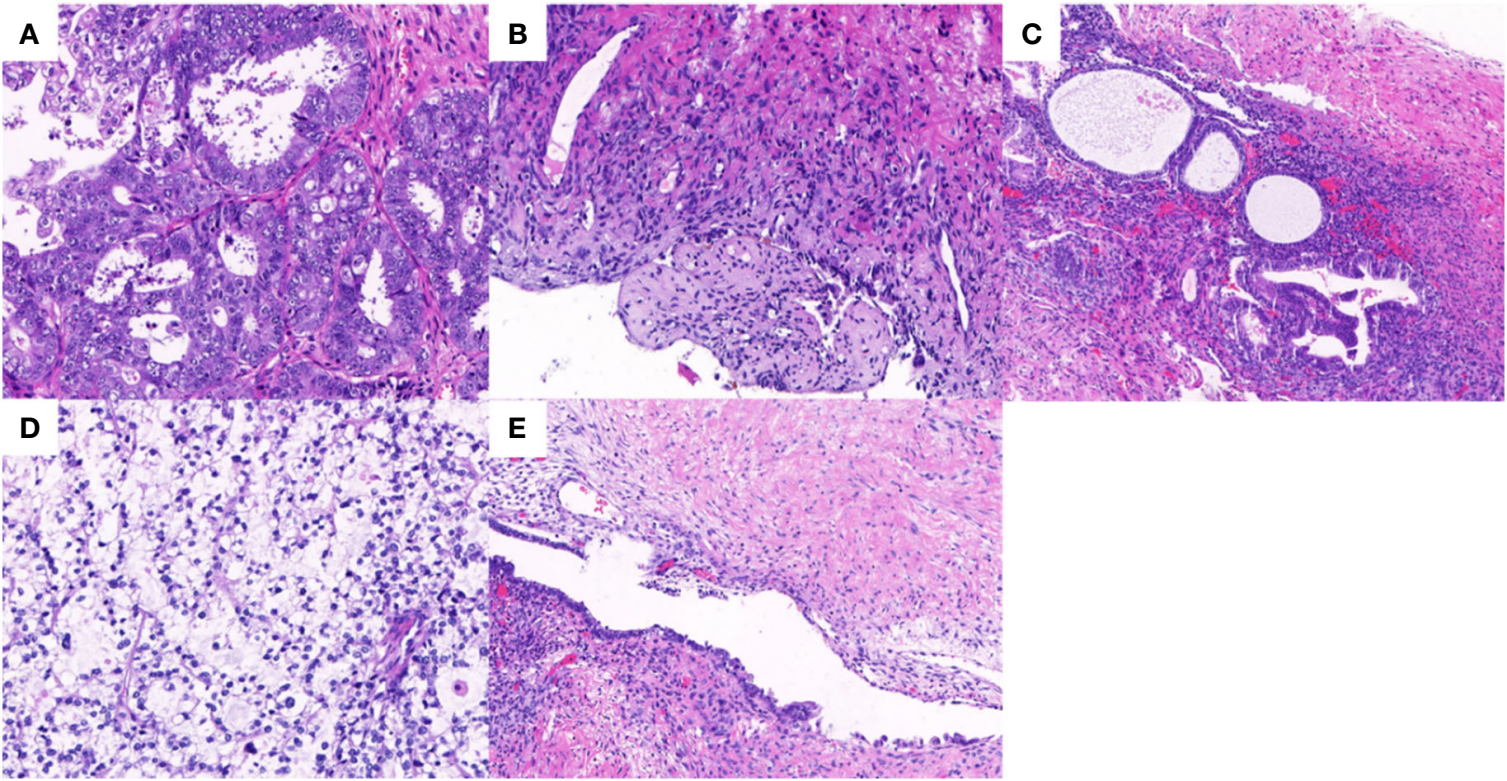
Hematoxylin-eosin staining in bilateral ovary tumors and endometrotic lesions. **(A)** The endometrioid adenocarcinoma in the left ovary with grade 2 (×20); **(B)** The borderline endometrioid adenocarcinoma area in the left ovary (×10); **(C)** The endometriotic area and atypical endometriotic area in the left fallopian tube (×10); **(D)** The clear cell carcinoma in the right ovary (×20); **(E)** The endometriotic lesion in the right ovary (×10).

**Figure 3:**
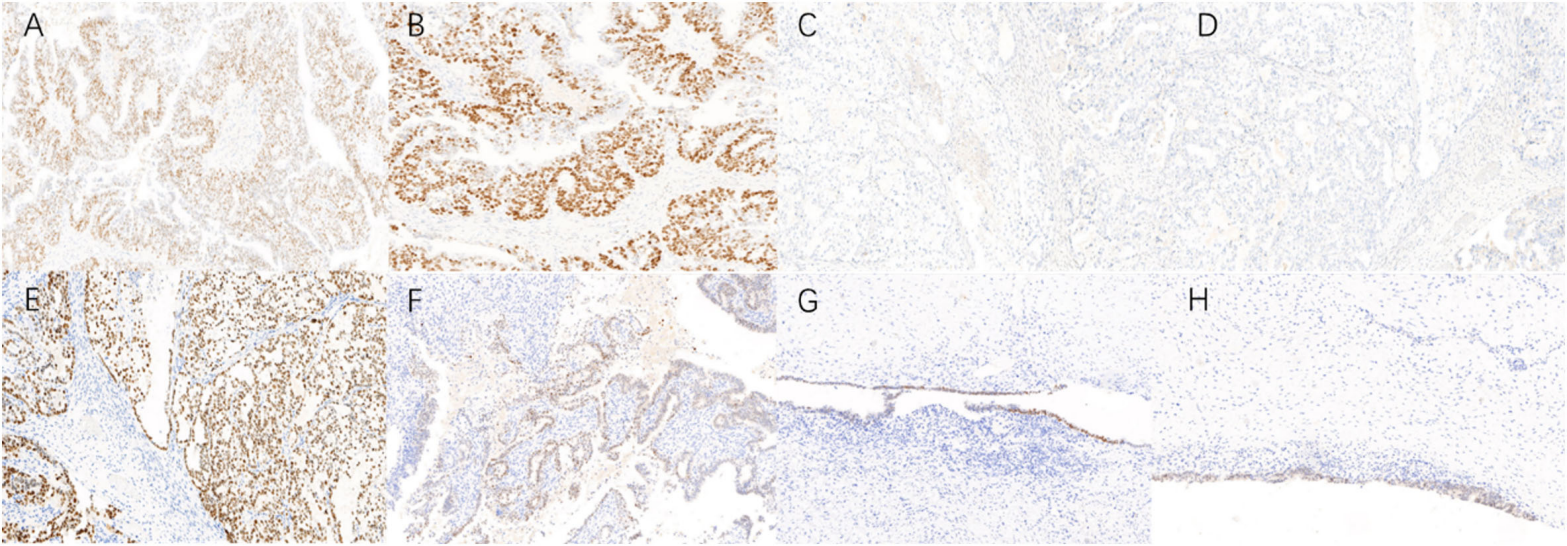
Immunohistochemistry (IHC) in bilateral ovary tumors and endometrotic lesions (×10). **(A, B)** Endometrioid ovarian cancer cells exhibited positive monoclonal expression of ER (+60%) and PR (+60%); **(C, D)** Clear cell ovarian cancer cells have no expression of ER, PR; **(E)** Clear cell ovarian carcinoma cells exhibited strong positive HNF-1β expression; **(F–H)** Atypical endometriotic lesion, endometriotic lesions of left ovary and right ovary also showed positive expression of HNF-1β.

The final paraffin pathology report confirmed malignancy in the bilateral ovaries, without infiltration of the uterus, fallopian tube, omentum, or lymph nodes. Lymphatic vascular involvement was negative. The left ovarian mass was consistent with ovarian endometrioid carcinoma that was moderately differentiated with large areas of necrosis, and the surrounding glands showed atypical endometriotic lesions and endometriotic lesions (**Figures 2A, B**). Ectopic endometrial glands and mesenchymal components were observed within the localized wall of the left fallopian tube tissue, consistent with endometriosis with focal ectopic glands with atypia (**Figure 2C**). The right ovarian mass was consistent with ovarian clear cell carcinoma and was surrounded by endometriotic cysts and corpus luteum cysts with haemorrhage (**Figures 2D, E**). Immunohistochemistry (IHC) was conducted to provide confirmation of the diagnosis. The tumour cells in the left EOC showed positive monoclonal expression of estrogen receptor (ER) (+60%) and progesterone receptor (PR) (+60%), along with patchy expression of monoclonal p16 and p53. However, the expression of ER and PR were negative in the right CCOC. While, the expression of p16, and p53 was absent consistent with left EOC. The Ki-67 labelling index was approximately 50% in the left ovary and 40% in the right ovary. In particular, hepatocyte nuclear factor 1 beta (HNF-1β) showed strong positive expression in the CCOC, positive expression in the atypical endometriotic lesions of the left ovary, and patchy expression in the endometriotic lesions of both ovaries (**Figure 3**).

**Figure 4:**
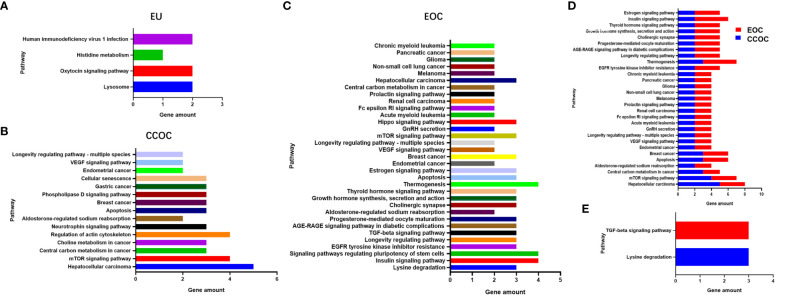
Pathway enrichment analysis revealed different gene pathways in eutopic endometrium **(A)**, clear cell ovarian cancer **(B)** and endometrioid ovarian cancer **(C)**. Twenty-nine gene pathways were identified enriched in both clear cell ovarian cancer and endometrioid ovarian cancer **(D)**. The TGF-β signaling pathway and lysine degradation pathway were uniquely enriched in endometrioid ovarian cancer and clear cell ovarian cancer, respectively **(E)**.

To explore potential aetiologies and therapeutic targets, whole-exome sequencing with next-generation sequencing was performed on eutopic endometrium (EU), bilateral tumours, and plasma samples. DNA sequencing results revealed somatic mutations in 29 genes for the EU, 66 genes for the CCOC, and 82 genes for the EOC (**Supplementary Table S1**). The shared mutated genes between the CCOC and EOC included ARIDIA, CCDC137, KHDRBS1, KRAS, PCDHB12, PIK3CA, SLC28A3 and ING1. ING1 was the sole gene mutated across all three samples (**Supplementary Figure S1**). Furthermore, NGS data analysis revealed microsatellite-stable status across all samples, with a low tumour mutational burden. Notably, only the EOC sample had a remarkably high homologous recombination deficiency score (50). Pathway enrichment analysis revealed different gene pathways for the three samples (**Figures 4A-C**). Twenty-nine gene pathways involving oestrogen metabolism, age, angiogenesis, apoptosis, and tumorigenesis, among others, were identified as enriched in both the EOC and CCOC samples (**Figure 4D**). The TGF-β signalling pathway and lysine degradation pathway were uniquely enriched in the EOC and CCOC, respectively (**Figure 4E**). Pearson correlation coefficients for signature features between the EU and CCOC were 0.9364 (P<0.0001), between the EU and EOC were 0.816 (P<0.0001), and between the EOC and CCOC were 0.8852 (P<0.0001) (**Supplementary Figure S1**).

## 3 Discussion

Endometriosis is characterized by the presence of endometrial tissue outside the uterine cavity, predominantly found within the pelvic cavity, ovary and fallopian tubes (1). Although it is a benign disease, it shares certain characteristics with cancer, such as local and distant invasion, resistance to apoptosis, and the ability to induce angiogenesis. Overall, the aetiology of this disease remains enigmatic (5). The pathogenesis of ovarian endometriomas is also a topic of debate, as no single theory can comprehensively explain the histogenesis of endometriosis, and a contradiction between the implantation theory and the metaplasia theory persists.

The association between endometriosis and ovarian cancer was initially described in 1925 by Sampson (6). This was further substantiated by Scott in 1953, who observed the presence of benign endometriosis near ovarian cancer (7). Although transformation of endometriosis to malignancy is a rare occurrence, with an estimated incidence between 0.7% and 1.6% among women (3), recent evidence suggests that these data might be underestimated. CCOC and EOC are the two histotypes most consistently linked to endometriosis (8). Concurrent endometriosis has been observed in approximately 21%–51% of women with CCOC and in 23%–43% of women with EOC (9).

The exact pathways leading to malignant transformation of endometriosis remain elusive. Accumulated evidence shows that the process of endometriosis-associated ovarian carcinogenesis is intricate and involves multiple stages. The implanted ectopic endometrium accumulates key mutations over time, progressively undergoing genetic and epigenetic alterations. This transformation is further promoted by the inflammatory and hyperoestrogenic microenvironment, coupled with the oxidative stress present within the endometriotic lesion (10). Recurrent point mutations are restricted to few typical oncogenes and tumour suppressors. The most frequently observed oncogene mutations shared by both histotypes are ARID1A, PIK3CA and PTEN (11–13). However, an increasing body of evidence from clinicopathological studies suggests that distinct pathways might be involved in malignant degeneration of endometriosis leading to CCOC and EOC, which suggests that the relationship between these histotypes and endometriosis might be different (4).

The majority of researchers agree on the existence of a dichotomy in the aetiology of the two different ovarian tumours correlating with endometriosis. At present, two main mechanisms are proposed to explain the dichotomy aetiology. In an interesting paper, Kajihara et al. observed positive expression of HNF-1β during the late secretory or menstrual phase in EU, endometrioma and endometriosis-associated CCOC, which was absent in endometriosis-associated EOC and ovarian cortical inclusion cysts. Therefore, they proposed the theory that endometriosis-associated clear cell carcinoma originates from the HNF-1β-positive eutopic endometrium retrogradely transported via menstruation. However, endometrioid histology involves transformation from inclusion cysts to a Müllerian epithelium as a precursor for endometrioid tumour development (14). In a recent retrospective analysis by Bergamini and coworkers, comparison between endometriosis-associated CCOC and EOC patients revealed distinct clinical characteristics (15). For example, women with endometriosis-associated endometrioid ovarian cancer were significantly younger at diagnosis and exhibited lower disease stages, a lower prevalence of high-grade tumours, and a higher probability of simultaneous endometrial carcinoma in the uterus. Accordingly, they hypothesized that the original precursor of endometriosis-associated CCOC might be located in the endometrium, where an already mutated endometrial cell may lead to development of ovarian endometriosis via retrograde menstruation and that carcinogenesis for EOC might occur within uterine endometrial cancer.

In our study, distinct IHC staining biomarkers were indeed observed in the bilateral ovaries. ER and PR were strongly positively expressed in the EOC in the left ovary but absent in the CCOC in the right ovary. Conversely, HNF-1β was strongly positively expressed in the CCOC but absent in the EOC. Interestingly, HNF-1β also showed positive expression in the atypical endometriotic lesions and endometriotic lesions of both ovaries. To explore the key aetiological factors, we also sequenced the secretory endometrium tissue curettage from the uterus. Our gene sequencing results revealed different known cancer-associated mutations (CAMs) among the EU, CCOC, and EOC, with the number of CAMs progressively increasing. The overlapping genes between CCOC and EOC included ARIDIA, KRAS, PIK3CA, CCDC137, KHDRBS1, PCDHB12, SLC28A3 and ING1, most of which have been reported in previous research.

In addition, we noted that ING1 was the only gene shared across all three samples. The ING gene belongs to the tumour-suppressor gene family and has regulatory functions in cell proliferation, apoptosis and cell senescence (16). This family includes five members (ING1-5) that enhance p53 activity by inducing acetylation or increasing its stability (17). ING1 has been demonstrated to be a tumour suppressor in a variety of human cancers, including lung cancer (18), colorectal cancer (19), and prostate cancer (20). To date, no studies have established a direct correlation between ING1 and the incidence of ovarian cancer. Given that we found this gene for the first time in three related tissue species, it deserves subsequent deeper exploration.

In the endometrium, each menstrual cycle is analogous to classic tissue injury and repair, which includes inflammation and its resolution, angiogenesis, tissue formation and remodelling or re-epithelialization (21). Similar to EU, the ectopic endometrium (endometriotic lesion) sheds glandular epithelial cells during menstruation, but to a considerably lesser degree in endometriotic stromal cells. Based on their findings, Suda et al. proposed that endometrial cells already harbouring CAMs, which confer selective advantages, may retrograde and find ectopic sites conducive for their growth, thereby fostering endometriosis development (22). Based on the above evidence and our results, we hypothesize a monophyletic histogenesis in the aetiology of CCOC and EOC histotypes: endometrial glands possessing preexisting CAMs, potentially with selective advantages, can easily implant onto ectopic sites and undergo clonal expansion. The implanted ectopic endometrium faces a harsher microenvironment characterized by hyperoestrogenism, inflammation, and oxidative stress-individually and collectively mutagenic factors that generate a hotbed for DNA damage and subsequent CAMs. Hence, endometriotic lesions accumulate different and sufficient CAMs, ultimately driving the process of malignant transformation.

## 4 Conclusion

Endometriosis-associated ovarian carcinogenesis is a multistep process. Research is needed to advance understanding of the disease aetiology, identify risk factors, and develop early detection methods and effective targeted therapies. Here, we report the simultaneous presence of two different histotypes of ovarian endometriosis-associated cancer in bilateral ovaries. Based on our genetic analysis, we hypothesize that endometriosis-associated CCOC and EOC may have a monoclonal origin, providing insights into understanding the different biological mechanisms underlying carcinogenesis.

## Data availability statement

The original contributions presented in the study are included in the article/**Supplementary Material**. Further inquiries can be directed to the corresponding author.

## Ethics statement

The studies involving humans were approved by Department of Obstetrics and Gynecology, the Seventh Medical Centre of Chinese PLA General Hospital. The studies were conducted in accordance with the local legislation and institutional requirements. The participants provided their written informed consent to participate in this study. Written informed consent was obtained from the individual(s) for the publication of any potentially identifiable images or data included in this article.

## Author contributions

LZ: Data curation, Investigation, Writing – original draft. YL: Investigation, Writing – original draft. FL: Investigation, Writing – original draft. XJ: Formal Analysis, Writing – original draft. JX: Formal Analysis, Writing – original draft. SZ: Investigation, Writing – original draft. YM: Project administration, Writing – original draft. AL: Investigation, Methodology, Writing – review & editing. WY: Supervision, Writing – review & editing, Project administration.

## Supplementary material

Supplementary Figure 1
**(A)** Pearson correlation coefficients for signature features between eutopic endometrium (EU) and clear cell ovarian cancer (CCOC) stood at 0.9364 (P<0.0001), between EU and endometrioid ovarian cancer (EOC) at 0.816 (P<0.0001), between EOC and CCOC at 0.8852 (P<0.0001) (**Supplementary Figure S1**). **(B)** Different loci in EU, CCOC and EOC, some of them (like ZNF469, CORO2A, and ZNF185) are not oncogenes. **(C)** The shared genes in EU, CCOC and EOC.
