# Supplementary material for: Simultaneous occurrence of two distinct histotypes of ovarian endometriosis-associated cancer in bilateral ovaries: implications for monoclonal histogenesis from a case report
Source: Front Oncol. 2023 Nov 27;13:1280529. doi: 10.3389/fonc.2023.1280529 (PMC10711050; doi:10.3389/fonc.2023.1280529)
Supplement: Supplementary file 1 [file Table_1.docx]

**Supplementary Table S1** Somatic alteration genes in three different samples.

| **Eutopic endometrium** | **Clear cell ovarian cancer** | **Endometrial ovarian cancer** |
| --- | --- | --- |
| A1CF | ABCC6 | ABLIM2 |
| ADCK5 | ACBD3 | ACP5 |
| AP1S1 | ACKR3 | ADAMTSL1 |
| ARSC | AFF1 | ADCY10 |
| COL4A2 | ARHGAP35 | ADGRF5 |
| CORO2A | ARID1A | AMH |
| CYP2W1 | ASPHD2 | APCDD1 |
| DNAH7 | BRINP1 | ARHGAP31 |
| EEF2 | C1orf228 | ARID1A |
| ENO3 | C9orf66 | ATL3 |
| GIPC1 | CAPN1 | BMP6 |
| HDGFL1 | CCDC137 | BPTF |
| HNMT | CDH11 | BRCC3 |
| ING1 | CDK13 | C20orf194 |
| KLHL38 | COASY | C2orf70 |
| KMT2D | CORO2A | C6orf222 |
| LEMD3 | CST9L | CCDC137 |
| LRRC8A | DGKZ | CCDC88C |
| NLRX1 | DHX9 | CEP170B |
| PPP3CA | DOCK1 | CLUH |
| RUFY2 | EFCAB6 | DAB2IP |
| SLC39A1 | EPB41L1 | DEF6 |
| SMTN | FAM83E | DLX5 |
| TG | GUSB | EIF4E2 |
| TRIM16L | HAPLN3 | ESX1 |
| USP26 | HECW2 | FBXO34 |
| ZGPAT | IGFN1 | FGD1 |
| ZNF185 | ING1 | FRMPD3 |
| ZNF469 | KCNA6 | GALR2 |
|  | KHDRBS1 | GDI1 |
|  | KIF7 | GPLD1 |
|  | KIFC2 | GREB1 |
|  | KRAS | HELZ2 |
|  | LAMTOR3 | HERC2 |
|  | LOC100996693 | IL12RB2 |
|  | LRP5 | ING1 |
|  | MRPL12 | KCNE3 |
|  | MTHFD1 | KCNJ4 |
|  | MYRF | KHDRBS1 |
|  | NEU2 | KRAS |
|  | NKAIN4 | LINGO4 |
|  | NKPD1 | LRP8 |
|  | NTRK3 | MAN1B1 |
|  | NXPE4 | MCTP2 |
|  | PCDHB12 | MED30 |
|  | PCDHB4 | MFRP |
|  | PHYHD1 | MRM2 |
|  | PIK3CA | MRPS18A |
|  | PNCK | MSI1 |
|  | PTPRZ1 | MST1L |
|  | RILPL1 | MUC16 |
|  | S100A10 | NACAD |
|  | SLC12A6 | NLRP2 |
|  | SLC28A3 | NYNRIN |
|  | SMARCB1 | PAQR9 |
|  | STRC | PCDHA12 |
|  | SYTL2 | PCDHB12 |
|  | TENM1 | PGR |
|  | THEMIS2 | PHKA2 |
|  | TMEM62 | PIK3CA |
|  | TRIO | PLOD3 |
|  | TRPS1 | PLXNA3 |
|  | VPS8 | PRDM15 |
|  | WDTC1 | PRDM16 |
|  | ZNF185 | PRRC2B |
|  | ZNF469 | RAPH1 |
|  |  | RASD2 |
|  |  | RNASE3 |
|  |  | RPS6KL1 |
|  |  | SEC31A |
|  |  | SETD1B |
|  |  | SLC28A3 |
|  |  | SMAD7 |
|  |  | SUSD6 |
|  |  | TGM6 |
|  |  | THBD |
|  |  | TMX3 |
|  |  | TNK2 |
|  |  | YDJC |
|  |  | ZFP62 |
|  |  | ZFYVE19 |
|  |  | ZNF133 |
