# Supplementary figures and images for: Simultaneous occurrence of two distinct histotypes of ovarian endometriosis-associated cancer in bilateral ovaries: implications for monoclonal histogenesis from a case report
Source: Front Oncol. 2023 Nov 27;13:1280529. doi: 10.3389/fonc.2023.1280529 (PMC10711050; doi:10.3389/fonc.2023.1280529)

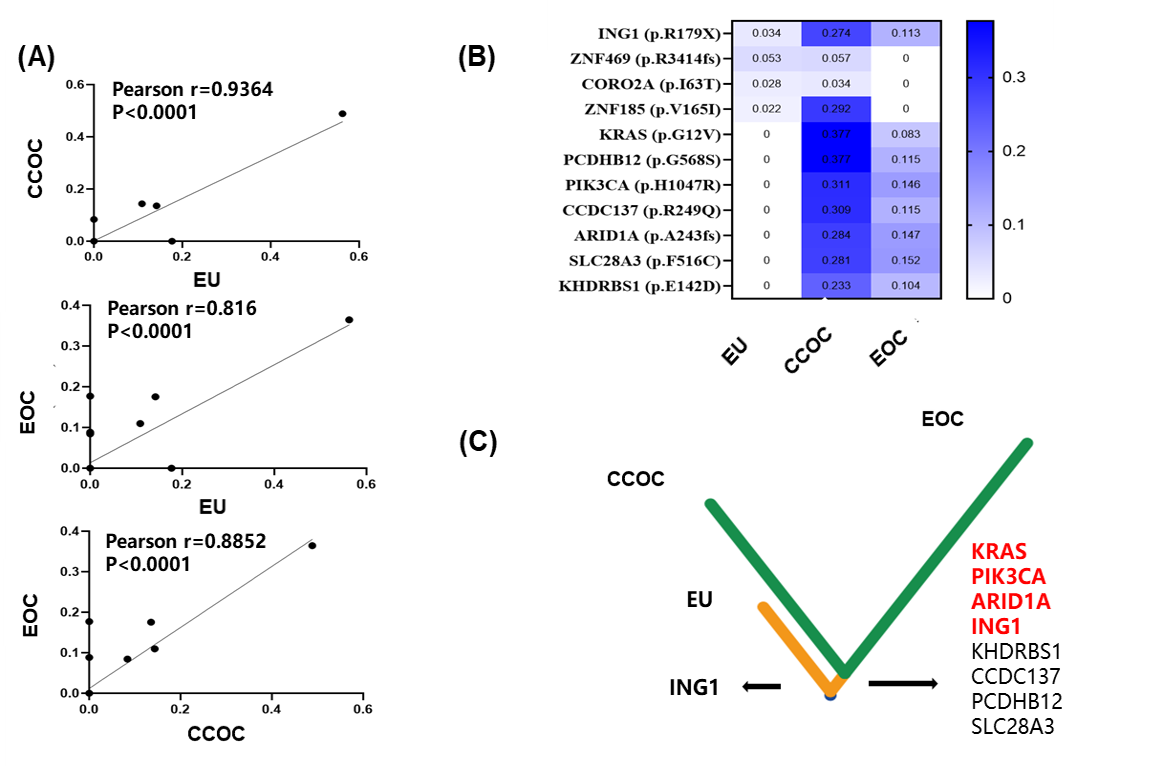

Supplement: Supplementary Figure 1 — (A) Pearson correlation coefficients for signature features between eutopic endometrium (EU) and clear cell ovarian cancer (CCOC) stood at 0.9364 (P<0.0001), between EU and endometrioid ovarian cancer (EOC) at 0.816 (P<0.0001), between EOC and CCOC at 0.8852 (P<0.0001) ( Supplementary Figure S1 ). (B) Different loci in EU, CCOC and EOC, some of them (like ZNF469, CORO2A, and ZNF185) are not oncogenes. (C) The shared genes in EU, CCOC and EOC. [file Image_1.tif]
